# Programming the Infant Gut: How Maternal and Early Life Nutrition Shape the Infant Microbiome and Long‐term Health—A Narrative Review

**DOI:** 10.1002/mnfr.70385

**Published:** 2026-01-19

**Authors:** Fanette Fontaine, Sondra Turjeman, Meriam Haib, Maria Carmen Collado, Karel Callens, Omry Koren

**Affiliations:** ^1^ Université Paris Cité Paris France; ^2^ Azrieli Faculty of Medicine Bar‐Ilan University Safed Israel; ^3^ Institute of Agrochemistry and Food Technology‐National Research Council (IATA‐CSIC) Paterna Valencia Spain; ^4^ Food and Agriculture Organization of the United Nations Via Delle Terme Di Caracalla Rome Italy; ^5^ Kyung Hee University Seoul The Republic of Korea

## Abstract

Childhood malnutrition, including undernutrition, obesity, and micronutrient deficiencies, remains a major global health burden. Emerging evidence points to the gut microbiome as a critical mediator linking maternal, prenatal, and early‐life nutrition to long‐term offspring health outcomes. From conception and through the first years of life, maternal diet, metabolic state, and environmental exposures shape offspring microbial colonization and maturation. Breastfeeding and consumption of fiber‐rich and fermented foods (maternal and post‐weaning) support beneficial microbiota, while high‐fat, high‐sugar diets, xenobiotics, and artificial additives may promote dysbiosis. The composition and diversity of the infant microbiome influence immune, metabolic, and neurodevelopmental processes and may also contribute to the intergenerational transmission of malnutrition. While commercial formulas increasingly include “biotics” to mimic human milk, exclusive breastfeeding remains the gold standard. Complementary feeding practices, including timing and diet quality, are known to modulate microbial maturation. Diet‐based interventions in pregnancy show promise in improving microbiome function and preventing disease in offspring. Because the microbiome is highly plastic in the first years of life, this window offers unique opportunities for preventive strategies targeting maternal and child nutrition. Integrating microbiome science into public health and dietary guidelines could enhance current approaches to breaking the cycle of malnutrition and promoting lifelong health.

## Graphical abstract

Good nutrition in pregnancy and early life plays a key role in shaping the offspring gut microbiota, which supports healthy growth, immune maturation, and neurodevelopment. Poor maternal and early‐life diets can disrupt this process, increasing the risk of obesity, undernutrition, and disease later in life. Breastfeeding, a fiber‐rich maternal diet, and careful feeding choices, including “biotic” interventions, can help build a healthy early‐life microbiota toward preventing lifelong health problems.

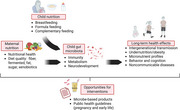

## 1 Introduction

Malnutrition in children, encompassing undernutrition, micronutrient deficiencies, and increasingly, early‐onset overweight, obesity, and noncommunicable diseases (NCDs), remains a critical global health challenge, contributing substantially to childhood morbidity, impaired development, and long‐term disease risk. In 2022, about 149 million children under the age of 5 exhibited stunted growth (too short for age), an estimated 45 million were wasted (too thin for height), and 37 million were overweight [1]. Women and children are particularly at risk of malnutrition [2], with both high‐and low‐income countries affected by at least one form of malnutrition, and some regions experiencing a double burden of malnutrition [2].

The biological roots of childhood malnutrition include not only the interplay of insufficient or excess nutritional intake and changes in energy expenditure, but also the less understood elements of genetics, epigenetics, the *in‐utero* environment, and the gut microbiome. Accumulating research supports that the gut microbiome is intimately involved in weight gain and loss, as well as in overall host homeostasis [3, 4]. The involvement of the microbiome in host health ranges from its role in obesity, undernutrition, cardiovascular diseases, and type 2 diabetes (T2D), as well as autoimmune and neurodegenerative diseases [5, 6, 7, 8, 9, 10, 11, 12], and data also suggest that the microbiome is involved in undernutrition in young children [13]. The human microbiome is shaped by multiple environmental factors and host genetics [14, 15, 16, 17, 18], and diet is one of the most influential factors [18, 19, 20, 21, 22, 23].

The period from conception to 2‐3 years of age, often referred to as the first 1,000 days of life, is a critical period for child development and health programing. The establishment and development of the gut microbiome during this period has effects on metabolism, the immune system, the nervous system, and behavior. Inappropriate development during this period might compromise subsequent attempts to fight obesity and NCDs in adulthood [24]. Data also suggests that the microbiome could be involved in the transgenerational transmission of malnutrition [20, 25, 26, 27]. Thus, optimizing microbiome acquisition and maturation should be considered in holistic preventive approaches to fight all forms of malnutrition and NCDs [28].

In this narrative review, we provide a broad synthesis of current evidence on how maternal and early‐life nutrition influence the developing infant's gut microbiome during the first 1,000 days of life. We aimed to identify emerging patterns and gaps in the literature, integrating findings across experimental models and human studies to highlight implications for long‐term health and inform future preventative interventions. Table 1 summarizes reviewed research, highlighting key characteristics of each study.

**TABLE 1:** Maternal diet during pregnancy.^*^ ^.^

Human or mouse	Geographic location	Study type	Sample Size^**^	Major findings	Ref.
Human	Europe	Meta‐analysis	16,295	Healthier, anti‐inflammatory maternal diets may lower childhood obesity risk	[62]
Human	USA	Prospective	33	Higher maternal adherence to a Mediterranean‐type diet was associated with favorable neonatal gut microbiome features and distinct DNA methylation signatures in cord/placenta tissue	[63]
Human	Australia	Prospective	213	Higher maternal prenatal gut‐microbiota diversity and healthy diet predicted fewer internalizing behavioral problems in her child	[64]
Human	Australia	Prospective	316	Higher maternal fiber and fat intake were jointly associated with lower infant allergy risk	[70]
Human	Taiwan	Cross‐sectional observational	39	Maternal intake of fruit and vegetables was associated with changes in neonatal gut‐microbiome composition	[71]
Human	Zimbabwe	Observational	207	Maternal microbiomes dominated by resistant‐starch degraders may contribute to birth‐weight outcomes	[72]
Mouse	—	Mechanistic	—	Low fiber in pregnancy increases offspring metabolic‐syndrome susceptibility, while SCFAs are restorative	[39]
Human; Mouse	China	Cross‐sectional; mechanistic	744	High‐fiber diets counteract maternal‐obesity‐linked cognitive and social deficits in offspring through microbiota‐gut‐brain pathway	[73]
Human	Tanzania	Intervention: probiotic	56	Maternal probiotic yogurt shifted infant, but not maternal, microbiota	[77]
Human	China	Prospective: probiotic	2731	Maternal yogurt intake in late pregnancy reduced infant eczema risk and recurrence at 3–6 months	[79]
Human	Japan	Prospective: fermented food	72624	Maternal fermented food, especially miso, intake was linked to longer infant sleep at 1 year	[80]
Human	Japan	Descriptive, Prospective: probiotic	9635	Collected baseline data on mothers and infants as part of Japan's national birth cohort; infant characteristics matched national averages	[81]
Mouse	—	Experimental: HFD diet	—	Maternal HFD disrupted offspring gut microbiota and social behavior; probiotics restored both	[83]
Primates (monkeys)	Japan	Experimental: HFD diet	—	Maternal HFD altered the gut microbiota of mothers and offspring; effects persisted after weaning, indicating that diet drives microbiome changes	[84]
Human	USA	Prospective: HFD diet	26	Maternal HFD reduced *Bacteroides* in infant guts at birth and 6 weeks, independent of maternal obesity	[85]
Mouse	—	Experimental:‐ HFD diet	—	Maternal HFD caused dysbiosis, weakened gut barrier, and greater colitis severity in offspring	[87]
Mouse	—	Experimental: HFD diet	—	Maternal HFD caused sex‐specific microbiome changes and worsened metabolic outcomes in male offspring	[88]
Mouse	—	Experimental: HFD diet	—	Maternal HFD altered microbiota, changed fetal brain metabolism, and caused anxiety‐like behavior in offspring	[89]
Human	USA	Prospective: observational	145	Maternal diet affected the infant gut microbiome at 6 weeks; effects differed by delivery mode (vaginal vs C‐section)	[90]
Human	USA	Prospective: observational	114	Maternal fish intake promoted a healthier, *Bifidobacterium*‐rich infant gut profile	[91]
Human	Spain	Observational	86	Maternal diet created two microbiota clusters that affected neonatal microbiota and infant growth	[92]
Human	Burkina Faso	Experimental	152	Balanced energy protein supplementation changed maternal and infant gut microbiome and improved infant growth	[53]
Mouse	—	Experimental	—	Maternal iron deficiency altered offspring gut microbiota and caused learning‐memory deficits	[94]
Human	Malawi	Experimental	695	Maternal LNS supplementation altered gut microbiome composition and function in mothers and accelerated infant microbiome maturation	[95]
Human	Netherlands	Prospective	913	Higher maternal vitamin D status was associated with lower *Bifidobacterium* counts and higher *Bacteroides fragilis* counts in 1‐month‐old infants	[97]
Human	Canada	Prospective	1157	Vitamin D supplementation did not affect *Clostridioides. difficile* but altered infant gut microbiota	[98]
Human	China	Prospective	87	Maternal vitamin D insufficiency was linked to lower infant gut microbial diversity at 6 months	[99]
Mouse	—	Experimental	—	Probiotic or prebiotic therapy prevented maternal high‐fructose–induced hypertension in offspring	[100]
Human	Canada	Case–control within prospective	100	Maternal artificially sweetened beverage consumption was associated with altered infant gut microbiota and higher BMI at one year	[101]
Mouse	—	Controlled experimental	—	Maternal P80 exposure disrupted gut microbiota and increased obesity risk in offspring	[103]
Human	China	Prospective	578	Prenatal PM2.5 exposure altered infant gut microbiota	[104]
Mouse	—	Controlled experimental	—	Perinatal TiO_2_ exposure impaired gut microbiota and intestinal immunity	[105]
Human	China	Prospective	578	Prenatal PM2.5 exposure altered infant gut microbiota	[114]
Human	Denmark	Prospective	75	Cesarean delivery altered infant gut microbiota composition	[115]
Mouse	—	Controlled experimental	—	Maternal AFB1 exposure impaired offspring gut immunity and microbiota composition	[116]
Mouse	—	Cross‐sectional; mechanistic	—	Maternal glyphosate exposure induced autism‐like behavior and gut microbiota changes in offspring, prevented by sEH inhibition	[117]

* The search strategy combined terms related to maternal and infant gut microbiome, nutrition, and infant health. The following keyword groups were used in various combinations in PubMed database: Microbiome: “Maternal microbiome”, “infant microbiome”, “child microbiome”; Nutrition: “breastfeeding”, “infant formula”, “nutrition”, “dietary fiber”, “high fat diet”, “micronutrient”, “vitamins”, “protein”, “sugar”, “artificial sweetener”, “food additives”, “probiotics”, “fermented food”, “xenobiotic”, “prelacteal food”, “undernutrition”; Infant/ child health status: “infant health”, “obesity”, “malnutrition”, “undernutrition”. In addition to the database search, the reference lists of all eligible studies were screened manually to identify additional relevant publications. Studies were included if they met the following criteria: peer‐reviewed original research or review articles; examined impact of nutrition on maternal and infant microbiome; investigated the link of microbiome changes with infant/ child health status. Studies focusing solely on nutrition and health without microbiome data were excluded. The selection and synthesis of literature were guided by the review's objective to map existing knowledge and highlight knowledge gaps.

** For human studies: The minimum number of dyads is given based on the number of samples reported for mothers and/or offspring.

## 2 Maternal Factors Shaping the Infant Gut Microbiome: Pre‐Pregnancy, Pregnancy, and Delivery

Although the fetal environment is sterile at birth [29, 30, 31, 32, 33], maternal microbial metabolites can reach the fetus via the placenta and exert developmental effects long before delivery. The maternal gut microbiota produces a variety of metabolites [34] that enter the maternal circulation and reach the fetal environment [35, 36, 37]. These microbial products may act directly or via epigenetic modifications, including changes in global histone acetylation and methylation in both mother and fetus [38, 39, 40].

At delivery, the neonate is colonized by maternal microbes, primarily those from the maternal gut and vaginal tract [41]. Any disruption in maternal microbial composition may therefore shape the initial microbiome of the infant and influence immune system development. Increasing evidence indicates that maternal health and nutrition exert a dual influence: they affect the mother's own microbiome and, in turn, that of the infant, with consequences for health across the life course.

### 2.1 Diet‐related Maternal Health Conditions

Pregnant women affected by diet‐related conditions, such as obesity, excessive gestational weight gain (GWG), gestational diabetes mellitus (GDM), or undernutrition, harbor a distinct gut microbiome compared to metabolically healthy women [22, 42, 43, 44, 45, 46, 47]. This altered maternal microbiota is transferred to the infant and persists for several months postnatally [48, 49, 50, 51, 52, 53]. Microbial alterations associated with obesity, GDM, or undernutrition during pregnancy may thus contribute to the intergenerational transmission of metabolic diseases, including obesity and T2D [49, 54, 55, 56, 57, 58], as well as neurodevelopmental abnormalities [59, 60]. Furthermore, these conditions are linked to increased risk for pregnancy complications such as preeclampsia, preterm birth, and GDM itself [61].

### 2.2 Maternal Diet

The composition, diversity, and quality of maternal diet before and during pregnancy are key modulators of infant developmental programming and microbiome colonization. In human studies, poor nutritional quality or extreme nutrient intake (either deficient or excessive) is associated with increased risk for NCDs in offspring [62]. Conversely, high adherence to a Mediterranean diet during pregnancy has been linked to greater abundance of short‐chain fatty acid (SCFA)‐producing microbes in neonatal meconium, as well as beneficial DNA methylation profiles in cord blood [63]. Similarly, findings from the Barwon Infant Study suggest that maternal diet indirectly reduces risk of behavioral disorders in children through increased maternal microbial alpha diversity [64]. Meta‐analyses further support that high‐quality diets during pregnancy may reduce risks of low birth weight and prematurity [65], both of which have been independently associated with disrupted infant microbiomes [66, 67, 68]. Maher et al. [69]. showed that, overall, the most significant nutrients associated with the gut microbiota composition of both infants and mothers were high‐fat diets, fat‐soluble vitamins, and fiber consumption.

Fiber intake. Fiber intake, a cornerstone of a healthy diet, promotes colonization by SCFA‐producing microbes and enhances microbial diversity. Human studies demonstrate that higher maternal fiber intake correlates positively with microbial diversity in both mother and infant [69]. Observational studies from Taiwan and Australia reveal that maternal consumption of fruits, vegetables, fiber, and fat is linked with beneficial infant microbiota profiles and reduced risk of food allergy, especially among women with *Prevotella copri* (recently renamed *Segatella copri*) enrichment [70, 71]. In rural Zimbabwe, enhanced microbial pathways for fiber degradation were positively correlated with neonatal growth [72]. Animal studies corroborate the protective role of maternal fiber intake, linking SCFA production to improved immune and metabolic health in offspring [39, 73]. However, responses to dietary fiber appear to be highly individualized, depending on the presence of fermenting keystone taxa [74]. Importantly, these benefits are most pronounced with long‐term adherence to a fiber‐rich diet initiated before conception, as short‐term changes during pregnancy may not suffice to substatially modulate microbial communities.

Probiotics and fermented foods. Probiotic supplementation during pregnancy is emerging as an effective strategy to modulate the maternal and infant microbiomes [75]. A meta‐analysis of 219 clinical trials found that maternal probiotic intake was associated with reduced risk of allergic outcomes in infants [76]. In Tanzania, the consumption of yogurt enriched with *Lacticaseibacillus rhamnosus* GR‐1 and Moringa increased infant fecal *Bifidobacterium* while reducing pro‐inflammatory Enterobacteriaceae members [77]. Fermented foods more broadly, such as yogurt, miso, and cheese, may exert similar benefits, including reduced risks of atopic dermatitis (AD) and food protein‐induced allergic proctocolitis [78, 79, 80, 81]. Moreover, in animal models, maternal consumption of fermented diets promoted neonatal *Lactobacillus* colonization and protected against intestinal inflammation [82].

High‐fat diets. A maternal high‐fat diet (HFD) can disrupt gut microbiome composition in both mother and offspring in mice, primates, and humans [69, 83, 84]. In human infants, maternal HFD exposure is linked to decreased offspring microbial diversity and depletion of *Bacteroides*, which may impair early immune training and nutrient metabolism [85, 86]. Animal studies report multiple adverse effects, including low‐grade inflammation, gut barrier dysfunction, fatty liver, and even behavioral changes in offspring [83, 87, 88, 89]. Nonetheless, the source of fat appears to be an important modifier, and emerging evidence suggests that deleterious effects may be mitigated by overall diet quality and specific dietary components [73, 83].

Protein sources. The effects of maternal protein intake on the offspring microbiome remain underexplored; however, initial findings indicate that protein source matters. In one human study, maternal dairy intake was positively associated with *Clostridium* spp. and *Staphylococcus*, while red and processed meats correlated with *Bifidobacterium* [90]. Birth mode also influenced these associations [90]. In another cohort, maternal fish consumption was linked to an infant gut microbiome enriched in *Bifidobacterium*, whereas absence of fish intake was associated with *Escherichia* dominance [91]. High intake of animal protein and saturated fat has been associated with increased child BMI z‐scores at 18 months, compared to diets richer in plant‐based proteins and fiber [92]. Balanced energy‐protein supplementation, as studied in Burkina Faso, significantly shifted both maternal and infant microbiomes and improved birth outcomes and infant growth via specific microbial pathways [53].

Micronutrients and vitamins. Micronutrient deficiencies can impair fetal development, and some effects appear to be mediated through the gut microbiome. Animal studies demonstrate that maternal deficiencies in iron, zinc, magnesium, folate, and vitamins A and D can disrupt the composition of maternal and infant microbiota [93, 94]. In humans, the effects of lipid‐based nutritional supplements (LNS) during pregnancy and lactation on the infant gut microbiota were tested in a large randomized clinical trial (RCT) conducted in Malawi. Infants 18 months of age from the maternal LNS‐treated group showed higher alpha diversity compared to infants from mothers supplemented with multiple conventional micronutrients, or iron and folic acid, but there was no effect on newborn microbiome maturation [95]. In addition, in human cohorts, prenatal vitamin D has been associated with lower abundance of potentially pathogenic bacteria such as *Clostridioides difficile* [96, 97, 98] as well as higher species richness overall [99]. In conclusion, in pregnant women at risk of micronutrient deficiencies, supplements are essential for normal fetal development, and some of the effects could be mediated by the microbiome, whose composition and function depend on micronutrient accessibility.

Sugar, artificial sweeteners, and other food additives. The western diet is characterized by significant consumption of dietary sugars such as fructose, but also by an abundance of food additives. Most dietary sugars are actively absorbed in the small intestine, but sugars can remain in the colon when overconsumed. In rats, a maternal high‐fructose diet was shown to induce hypertension by altering the gut microbiota in male offspring into early adulthood. Maternal probiotic (*Lacticaseibacillus*) consumption and prebiotic inulin intake during pregnancy and lactation are protective against this effect [100].

Some sweeteners (e.g., sucralose) are poorly absorbed in the small intestine, and a significant proportion of what is ingested reaches the colon. In a prospective Canadian cohort study, maternal consumption of artificially sweetened beverages during pregnancy was associated with modifications in both infant gut microbiota and metabolite profiles (higher succinate and spermidine). Shifts in microbial communities included depletion of Bacteroides spp., and enrichment in *Akkermansia muciniphila*. These changes were associated with higher infant BMI at one year [101], suggesting that infants exposed to artificially sweetened beverages during gestation may be at higher risk of microbiome changes related to early‐life predisposition to metabolic diseases.

In addition to artificial sweeteners, multiple other food additives could affect the gut microbiome [102]. In animal models, maternal exposure to emulsifiers such as polysorbate 80 (P80) led to gut microbiota shifts in offspring, with the increase of *Akkermansia* (a mucin‐degrading bacteria), *Prevotella*, and *Ruminococcus* and also a potentially harmful genus, *Helicobacter* [103]. Maternal exposure to emulsifiers predisposes offspring to obesity. Interestingly, in mice, some negative effects of P80 might be countered by supplementation of pectin [104], a soluble fiber found in fruits and vegetables. In mice, perinatal exposure to food‐grade titanium dioxide (fg‐TiO_2_), a common food additive, at realistic human doses, also led to alterations of gut microbiota composition and the intestinal barrier, and increased risk of food allergy in young males [105].

Xenobiotics. Food is a major source of exposure to various compounds such as mycotoxins, heavy metals, chemical residues such as pesticides, microplastics, and veterinary drugs. Increasing data shows that these xenobiotics could impair gut microbiome composition and function [106, 107, 108, 109, 110, 111, 112], and consequences of toxicity are expected to be more deleterious in prenatal and early‐life periods [113]. Very few studies have investigated the impacts of these compounds during the prenatal period on the microbiome of the mother or her infant. Most studies focus on animals. In mice, exposure to chemical polychlorinated biphenyls (PCBs) in the prenatal period via maternal diet caused significant mucosal barrier defects in the ileum and colon of offspring. PCBs exposure increased the intestinal inflammation profile and resulted in dysbiosis of gut microbiota in juvenile mice developmentally exposed to 1 mg/kg/d PCBs when compared to wild‐type controls [114]. Interestingly, probiotics might reduce the buildup and impact of certain contaminants. Consumption of yogurt supplemented with 10^10^ CFU L. rhamnosus GR‐1 had a protective effect against further increase in mercury and arsenic blood levels in at‐risk groups of pregnant women, but this effect was not significant in children [115].

In mice, maternal exposure to mycotoxins (aflatoxin B1) modifies offspring microbiome composition and decreases the ability to tackle intestinal pathogens through disturbed intestinal barrier homeostasis [116]. Similarly, offspring of dams exposed to the pesticide glyphosate presented autism spectrum disorder‐like behavioral abnormalities associated with microbiome and SCFA profile changes, compared to untreated control mice [117]. In humans, maternal exposure to other heavy metals, including arsenic and zinc, impacted infant microbiomes by reducing diversity (arsenic) and lowering *Bifidobacterium* abundance (zinc) [113]. Effects of such chemicals on the offspring microbiome are concerning for child health, but these studies mostly used doses of chemicals above acceptable daily intake levels, making it difficult to assess the effect of more common low‐level exposure [117]. In addition, most studies only assess one type of chemical, but it is probable that chemical exposure through food is not so binary and that some compounds might have synergistic effects.

Altogether, these data underscore the profound and multifaceted influence of maternal diet and environmental exposures on the infant gut microbiome. While many findings align with studies in the general population, specific responses during pregnancy and early life suggest heightened sensitivity or distinct mechanistic pathways. These intergenerational effects are further shaped by delivery mode, medication use, and postnatal nutrition. Understanding how these variables converge to shape the early‐life microbiome is crucial for developing targeted nutritional interventions aimed at improving lifelong health.

## 3 Infant Nutrition: From Birth to Weaning

### 3.1 Impact of Prelacteal Foods

Prelacteal feeding, the introduction of liquids or non‐breast milk foods before the onset of regular breastfeeding, is common in several low‐ and middle‐income countries and is associated with reduced durations of exclusive and overall breastfeeding [118, 119]. Despite its prevalence, the biological consequences of prelacteal feeding remain poorly understood. Early exposure to nonhuman milk foods may delay lactation [120]. Breastfeeding is a key driver of microbial colonization in early life [120]. Therefore, disruptions in the initiation or continuation of lactation, such as those caused by prelacteal feeding, can influence microbial colonization in early life and impair health outcomes. Preclinical studies in mice have demonstrated that deprivation of colostrum or delays in its intake can negatively affect infant growth, development, and microbial composition [121]. While human data are limited, these findings raise concerns about potential long‐term impacts on immunity, metabolism, and neurodevelopment.

### 3.2 Impact of Breastfeeding

A large body of evidence highlights the health benefits of breastfeeding, including enhanced protection against infections and reduced risk of obesity [122, 123], type 1 [124] and type 2 diabetes [125], asthma [126], and cardiovascular risk [127]. Longer and exclusive breastfeeding (EBF) durations amplify these benefits, and recent studies suggest that the gut microbiome may be a key mediator of these outcomes [128]. Clear differences exist between the microbiota of breastfed and formula‐fed infants [28, 129, 130, 131], and breastfeeding is considered one of the most influential factors shaping infant gut microbial structure [132, 133]. Typically, breastfed infants exhibit lower microbial diversity but an increased relative abundance of *Bifidobacterium* spp., including *B. breve*, *B. bifidum*, and *B. longum*, which are adapted to metabolize human milk oligosaccharides (HMOs) [133, 134, 135]. Meta‐analyses confirm that EBF infants have lower bacterial diversity, reduced Firmicutes and Bacteroidetes, and lower enrichment of carbohydrate metabolism pathways, while pathways related to lipid and vitamin metabolism are more prominent [130].

These functional differences are reflected in SCFA profiles: Formula‐fed infants exhibit higher overall SCFAs, particularly butyrate, while EBF infants have higher acetate levels driven by *Bifidobacterium* activity; mixed‐fed infants have relatively more propionate. Notably, these differences persist beyond six months [136]. Short‐term formula supplementation in the first days of life can also alter microbial development. Infants who received formula supplementation early, even if exclusively breastfed later, had lower Bifidobacteriaceae and higher Enterobacteriaceae levels at 3–4 months compared to EBF infants. Longer‐term formula use during the first six months of life further shifted the microbiota toward a profile typical of nonbreastfed infants [137]. In contrast, the introduction of complementary foods without formula maintained a microbiota closer to that of EBF infants. Functionally, the microbiota of formula‐fed infants more closely resembles adult microbial communities, with increased diversity and genes involved in bile acid metabolism and methanogenesis [138]. Breastfeeding may also mitigate dysbiosis in infants exposed to perinatal antibiotics, born by cesarean section, or born prematurely [139, 140, 141, 142].

Several elements of human milk (HM) shape the infant gut microbiota through complementary mechanisms. HMOs promote the growth of beneficial taxa like *Bifidobacterium*, while antimicrobial components, such as secretory IgAs, lactoferrin, IgGs, and defensins, suppress pathogenic colonization and modulate immune responses. These factors help establish a protective microbial ecosystem that supports early‐life immune and metabolic development. Importantly, both the composition and microbiome of HM are influenced by maternal factors, including health status and nutrition [143]. For example, HM of obese and overweight mothers had decreased *Bifidobacterium* and increased *Staphylococcus* [144]. Women with celiac disease on a gluten‐free diet also show alterations in HM microbiota, such as increased *Rothia mucilaginosa*, a species linked to autoimmune conditions [145]. While direct data are on how these milk microbiome changes affect infant gut colonization are still limited, the continued maternal‐infant microbial colonization underscores their potential relevance.

Maternal dietary habits have been shown to influence both her milk composition and her breastfed infant's microbiome and growth [146, 147]. For instance, high consumption of fruits and vegetables was associated with increased abundance of several HMOs in breast milk. Moreover, dietary components can be directly incorporated into milk oligosaccharides; Neu5Gc, a red meat‐derived compound, has been detected in HMOs and was positively associated with *Bacteroides* abundance in infant stool [148]. These findings reveal a clear mechanistic link between maternal diet, milk‐derived glycans, and infant microbial colonization.

The impacts of maternal probiotic supplementation during lactation have also been explored. A meta‐analysis of six studies found that probiotic use can lead to identification of the supplemented strains in the HM [149]. In most studies, Lactobacillus spp., administered during pregnancy and/or lactation, reduced *Staphylococcus* counts in HM of supplemented women [149]. Downstream benefits for infants have also been suggested, including better weight control [150], reduced risk of eczema [151], and potential improvements in cognitive development [152]. Although more studies are needed, these findings highlight the potential of maternal dietary and probiotic interventions during lactation as accessible strategies to beneficially shape the infant microbiome and long‐term health.

### 3.3 Infant Formula

While breastfeeding remains the gold standard for infant nutrition, commercial formula has undergone substantial modifications to more closely replicate the composition and functional impact of HM, particularly with respect to its influence on the gut microbiota. Most infant formulas are derived from cow's milk and are increasingly supplemented with bioactive compounds such as prebiotics, probiotics, synbiotics, and postbiotics, collectively termed “biotics” [153]. These innovations aim to narrow the microbiota gap between breastfed and formula‐fed infants.

Prebiotics are among the most studied formula additives. Chemically stable, low‐risk, and easy to administer, they selectively promote the growth of beneficial microbes [154]. Commonly used prebiotics include galacto‐oligosaccharides (GOS), fructo‐oligosaccharides (FOS), and pectin‐derived oligosaccharides. The best‐studied combination, a 9:1 mixture of short‐chain GOS (scGOS) and long‐chain FOS (lcFOS), mimics some of the structural functions of HMOs in breastmilk [154]. Supplementation with this mixture lowers fecal pH, promotes an SCFA profile similar to breastfed infants, and increases *Bifidobacterium* and *Lactobacillus* abundance [155, 156]. It is also associated with reduced colonization by opportunistic pathogens like *E. coli*, *Enterococci*, and *Clostridia* [138, 157].

HMOs themselves are increasingly being incorporated into infant formulas, although their complexity and manufacturing cost remain challenges. Two HMOs, 2′‐fucosyllactose (2′‐FL) and lacto‐N‐neotetraose (LNnT), are the most commonly added. In vitro and in vivo studies show that 2′‐FL supports Bifidobacteria, inhibits pathogen adherence, strengthens the intestinal barrier, and promotes mucosal immune function [153]. Other HMOs, such as 3‐Fucosyllactose, 6'‐Sialyllactose, and 3'‐Sialyllactose are also being used, and data from animal studies showed that HMOs could also prevent necrotizing enterocolitis (NEC) [158, 159].

Probiotic‐containing formulas have been available for over three decades, typically enriched with *Bifidobacterium* spp. and lactic acid bacteria such as *Lactobacillus* spp., both generally recognized as safe (GRAS) for infant use. However, outcomes vary widely depending on strain and host characteristics [160]. For example, supplementation with *L. rhamnosus* GG or a multi‐strain combination (e.g., *B. infantis* Bb‐02, *B. lactis* Bb‐12, and *S. thermophilus* TH‐4) has been linked to NEC risk reduction in preterm infants [160], but health benefits of probiotic‐supplemented formulas are very strain and host specific [161].

Synbiotics, combinations of live microbes and substrates that confer synergistic health effects, have also been tested in formula. A common example includes the 9:1 scGOS/lcFOS mixture paired with *B. breve* M‐16 V. This synbiotic promotes *Bifidobacterium* colonization in C‐section‐delivered infants, reduces potential pathogens, modifies SCFA profiles (e.g., increased acetate, decreased butyrate), and improves skin health by lowering atopic dermatitis symptoms [153, 162, 163, 164].

Fermented “postbiotic” infant formulas, which undergo lactic acid fermentation during production but contain negligible viable bacteria in the final product, are another approach to improving infant microbiome maturation and health outcomes. One such formula, fermented with *L. paracasei* CBA L74, was shown to promote immune maturation and microbiome development similar to breastfed infants [165]. Other fermented formulas containing B. breve C50 and S. thermophilus 065, with or without added prebiotics (scGOS/lcFOS (9:1)), were shown to increase *Bifidobacterium* spp. and decrease *C. difficile*, in addition to conferring health benefits [153].

Another nuance of formula milk is that it needs to be rehydrated, and exposure to domestic water has been correlated with changes in the microbiome. The type of water used, bottled or filtered, also differentially affects metabolic pathways [51]. These effects, although not yet fully characterized, may partially explain microbiota variation among formula‐fed infants.

For infants with cow's milk protein allergy, amino acid‐based formulas (AAF) and AAF‐synbiotic ones (AAF‐syn) have been developed. Compared to standard AAFs, synbiotic‐enriched formulations are associated with reduced microbial diversity and increased Bifidobacteria, closely resembling the microbiota of healthy EBF infants [165]. In contrast, soy‐based formulas appear to profoundly alter gut microbial composition, characterized by lower *Bifidobacterium* and higher Lachnospiraceae and Ruminococcaceae, along with enrichment in pathways linked to dysbiosis [51, 166].

In conclusion, most formulas supplemented with “biotics” are associated with positive microbiome changes, causing shifts in the infant gut microbiota toward that of breastfed infants. Specifically, an increase in *Bifidobacterium* and a decrease of potential pathogens such as *C. difficile* are observed. Consumption of optimized formulas might improve infant health compared to classical formula, but the bacterial compositions that they support do not yet fully approach an EBF‐derived microbiota.

### 3.4 Xenobiotics in Human Milk and Infant Formula

Both HM and infant formula can contain environmental contaminants, xenobiotics, originating from packaging, agricultural residues, or industrial processes. Microplastics, for instance, have been detected in every tested formula brand and in HM itself [167, 168]. Other concerning compounds found in HM include mycotoxins [169, 170], heavy metals [171, 172, 173], bisphenol A (BPA) [174, 175], and agricultural chemicals [176, 177], as well as antibiotic residues [178]. Despite growing concerns about their potential consequences for infant development and long‐term health, significant gaps remain in our understanding of the presence, sources, and health impacts of chemicals and other xenobiotics in HM and in infant formula. Further scientific evidence is needed to determine its impact on malnutrition and infant health.

## 4 Child Nutrition after Weaning

### 4.1 Introduction of Solid Foods and Food Diversification

According to WHO and UNICEF recommendations, solid foods should be introduced after six months of exclusive breastfeeding. In practice, however, solids are often introduced between four and six months of age. While some studies suggest that in EBF infants, the introduction of solids alone has only a marginal impact on gut microbiota composition [51, 137], the transition from milk to solid foods marks a key developmental milestone, triggering increased microbial diversity and a shift toward a more adult‐like microbiota. This includes an expansion of adult‐associated taxa, particularly from the Lachnospiraceae and Ruminococcaceae families, and a concurrent decline in and replacement of *Bifidobacterium* members [134, 179]. Interestingly, infants transitioning from breastfeeding to formula‐feeding exhibit similar patterns to those weaned onto solids, suggesting the strong role for milk withdrawal in shaping microbial succession [179, 180, 181]. Among formula‐fed infants, the introduction of solids has been associated with increased levels of *Streptococcus* and *Coprobacillus*, as well as a relative increase in *Bifidobacterium* [134].

The microbiome changes observed during weaning are also associated with functional maturation. Genes involved in the metabolism of complex carbohydrates, starches, plant‐derived polysaccharides, and xenobiotics become enriched, along with genes for vitamin biosynthesis [134]. SCFA profiles shift as well: In the early weaning period, there is a transition from high succinate and low acetate to elevated levels of lactate and formate. Complete cessation of breastfeeding is followed by increases in butyrate and propionate production, largely due to expansion of Clostridiales taxa [182]. Complementary foods rich in fiber and protein exert particularly strong effects on microbial diversity and interspecies interactions, facilitating cooperative degradation of resistant starches and plant polysaccharides [135].

### 4.2 Timing and Type of Complementary Foods

The timing of complementary food introduction plays a critical role in microbiome development. In a North Carolina, USA cohort, infants who began complementary feeding before three months had significantly higher microbial diversity and altered taxonomic profiles, including reduced *Bifidobacterium* and increased levels of *A. muciniphila*, *Bilophila wadsworthia*, Lachnospiraceae, and *Roseburia*, as well as increased SCFA concentrations. Some of these changes persisted up to one year [183]. Another prospective study showed similar results; introduction of complementary foods before four months resulted in faster gut microbiome maturation than introduction to complementary foods after six months of age [184]. However, other reports, such as that by Vacca et al., found no differences in microbial diversity between infants weaned before or after four months [185].

While high diversity is generally considered beneficial in adults, its interpretation in early infancy is more nuanced. Consequences of early weaning, like greater microbial diversity at three months, have been associated with an increased risk of being overweight later in life [137, 186], possibly due to premature depletion of beneficial Bifidobacteria, which are known to play a critical role in immune regulation [179, 183]. Furthermore, the introduction of solids before four months has been linked to a higher risk of childhood overweight, particularly in formula‐fed infants or those breastfed for less than four months. No such association was seen in infants breastfed for longer than four months, concomitantly with solid food introduction [187].

Specific food components may also exert distinct effects. For example, early introduction of gluten (by three to four months) has been associated with a reduced risk of celiac disease, while high levels of gluten exposure early in life may increase the risk of type 1 diabetes in humans [188, 189]. The precise role of the microbiome in these processes remains unclear but represents an important avenue for future research.

### 4.3 Sustainable Complementary Foods: The Case for Rice Bran

Ensuring access to safe, sustainable, and culturally appropriate complementary foods is particularly critical in regions experiencing food insecurity or political instability. An illustrative example comes from a study by Zambrana et al., which tested dietary rice bran supplementation during the weaning period (6–12 months of age) in infants from Nicaragua and Mali [190]. This intervention improved length‐for‐age z‐scores in both countries, and weight‐for‐age z‐scores significantly improved in the Malian cohort at 8 and 12 months. Rice bran is a practical dietary intervention strategy that merits development in rice‐growing regions that have a high prevalence of growth stunting due to malnutrition and diarrheal diseases. Furthermore, it is a very good example of optimization of all parts of the rice.

### 4.4 Iron Supplementation

Iron is a critical micronutrient in infancy, required for immune function, neurodevelopment, and overall growth. To prevent and treat iron deficiency anemia, fortification and supplementation strategies, including the use of iron‐containing micronutrient powders (MNPs), are widely implemented [191]. However, accumulating evidence from both animal [192] and human [193, 194] studies suggests that iron supplementation can significantly alter the gut microbiome and potentially exacerbate susceptibility to infections.

Iron supplementation has been shown to disrupt the balance of bacterial, fungal, and protozoan communities in the gut [193, 194]. In murine models, short‐term iron administration suppressed the virulence of *Citrobacter rodentium* by inducing insulin resistance and increasing intestinal glucose levels, paradoxically converting the pathogen into a commensal organism [192]. Yet, in most contexts, supplemental iron is associated with unfavorable microbial shifts. In both animals and humans, iron reduces the abundance of *Bifidobacterium* while enhancing the growth and virulence of enteric pathogens such as *Salmonella*, *E. coli*, *Shigella*, and *Campylobacter* [193, 195].

Evidence from studies in Kenyan infants confirms this risk: Iron supplementation led to increased colonization by *E. coli*, a reduction in beneficial Lactobacillaceae, and elevated markers of gut inflammation [196, 197, 198]. Yet these effects appear context dependent. A similar intervention in South African infants showed no detectable changes in microbiota composition, underscoring the potential influence of regional differences in environmental exposures, baseline nutrition, and hygiene practices [199]. Iron may also interact with other treatments, potentially diminishing their effectiveness. For instance, data suggest that iron fortification may reduce the efficacy of broad‐spectrum antibiotics against *E. coli* infections in children [200].

As we describe, although iron‐rich MNPs are effective at reducing anemia, they may increase gastrointestinal risks, particularly in low‐resource settings. To address this, newer formulations are being tested. These include combinations of iron with antioxidants such as vitamin E [201] or with probiotics, designed to mitigate adverse effects while maintaining efficacy [202]. A Kenyan trial found that co‐supplementing MNPs with prebiotic GOS mitigated most adverse effects. Notably, infants of nonsecretor mothers (lacking certain HMOs) responded better to GOS, highlighting the role of *Bifidobacterium* and the gut microbiota in modulating iron's impact [203, 204].

Together, these findings highlight the need for microbiota‐aware iron supplementation strategies in infancy, particularly in vulnerable populations where both infection risk and micronutrient deficiency burdens are high.

### 4.5 Child Nutrition Beyond the First Year of Life

While the effects of diet on the adult gut microbiome are well established [205], relatively fewer studies have examined these associations in infants and young children. Nonetheless, available data suggest that the microbial responses to specific foods and dietary patterns in children largely mirror those observed in adults [206]. Importantly, early‐life dietary habits may have long‐lasting effects on microbiome composition and host health.

In a recent clinical trial involving 394 healthy infants, those weaned onto a Mediterranean‐style diet, comprising fresh, seasonal foods, exhibited greater microbial diversity, increased abundance of beneficial taxa such as Coriobacteriaceae and *Lachnoclostridium*, and reduced levels of *Ruminococcus gnavus* by age four, compared to infants predominantly fed industrial baby food [207]. Similarly, findings from the Dutch KOALA Birth Cohort showed that breastfeeding duration and preschool dietary habits correlated with both the structure and functional capacity of the gut microbiome in children aged six to nine. Notably, children with *Bacteroides*‐ or *Prevotella*‐dominated microbiomes who consumed more dietary fiber had lower plasma insulin levels [208].

As in adults, specific food groups are associated with distinct microbial profiles in children. A study of one‐to two‐year‐olds found that unprocessed foods (e.g., meat, fish, fruits) were positively associated with *Ruminococcus*, *Bacteroides*, and members of the Lachnospiraceae family, while processed foods correlated with increased *Blautia* and *Clostridium* [209]. In Australian children aged 2–3, dairy intake was negatively associated with overall microbial diversity but positively linked with the Firmicutes:Bacteroidetes (renamed Bacillota and Bacteroidota, respectively) ratio and *Erysipelatoclostridium* spp. [210]. Vegetable intake correlated positively with *Lachnospira*, and consumption of soy, pulses, and nuts was associated with greater *Bacteroides xylanisolvens*, a next‐generation probiotic candidate due to its anti‐inflammatory potential [211]. Intriguingly, consumption of apples and pears was negatively associated with *R. gnavus*, a species implicated in inflammatory diseases including Crohn's disease [212].

The child microbiome does not stabilize until the age of three [180], and some data suggest that it continues to evolve until the age of five or beyond [135]. Despite gradual change and increased diversity in the first years of life, the juvenile microbiome has lower bacterial diversity, lower functional complexity, and a higher degree of interpersonal variation in diversity compared to adults [135]. Data suggest that the juvenile microbiome might be less resilient and that it demonstrates higher plasticity than adult microbiomes [213, 214, 215]. Adult microbiomes are relatively resistant to brief dietary interventions and are strongly shaped by long‐term adherence to consistent dietary habits [216]. On the other hand, in a small study, the gut microbiome of seven subjects (5 adults and 2 children) with urban lifestyles was measured after 2 weeks of immersion in an Amerindian village in the rainforest. Responses to the lifestyle and the traditional diet (low‐fat/high‐fiber unprocessed diet) were more pronounced in children than in adults [213]. In another study, one portion of almonds was added to the daily diets of adults and children, and induced changes in the gut microbiota were stronger in children than in adults [214]. These data suggest that the first years of life could provide opportunities for diet‐based, microbiota‐derived interventions to promote health or prevent microbiota dysbiosis.

Pro‐ and prebiotics could be used for dietary interventions with effects on health that are strain and context dependent. For example, in infants and children, probiotics reduce the risk of common acute infections [217], lower the incidence of AD and relieve AD symptoms. Interventions with mixed‐strain probiotics have enhanced preventive and curative effects [218]. However, another meta‐analysis based on 19 RCTs did not support recommendations to use probiotics in the prevention of asthma in infants [213]. Similarly, while a recent RCT showed some promise in modulating the preterm infant microbiota toward that of full‐term infants with multi‐strain probiotics, there was no protective effect against colonization by multidrug‐resistant organisms (primary study endpoint) [219].

In addition to probiotics, dietary intervention could be used to modulate the emerging microbiome and improve health. NCDs usually develop and are diagnosed after 3 years of age. Accordingly, in a clinical trial on obese children (3‐16 years old), a diet enriched in nondigestible carbohydrates (with a ready‐to‐use formula containing various prebiotics, whole grains, and traditional Chinese medicinal foods) changed the gut microbiota and reduced inflammation and weight gain. Metagenomic and metabolomic data showed a significant increase of functional genome group expression for acetate production from carbohydrate fermentation and a reduction of metabolites usually associated with adverse metabolic outcomes (trimethylamine N‐oxide and indoxyl sulfate) after dietary intervention compared to baseline measures before intervention. Fecal transplant of microbiomes from obese children before intervention into GF mice induced increased inflammation and larger adipocytes than transplant with the microbiome of the same individuals after intervention. This trial (and other research) demonstrated that microbiomes from obese individuals can transfer characteristics resembling metabolic syndrome; thus, the dysbiotic microbiome could be targeted with a dietary approach to improve health [220].

Several additional studies established links between diet, microbiome, and health outcomes in children. An analysis of 361 children at 3 years of age from the study of the Vitamin D Antenatal Asthma Reduction Trial in the USA revealed interrelationships between diet, microbiome, and asthma. Children who were breastfed for more than 4 months had a lower incidence of asthma by the age of 3. Metabolomic analysis identified a causal pathway whereby intestinal metabolites mediated part of the association between fried and processed meat consumption and asthma, with the family Christensenellaceae and specific plasma metabolites positively associated with both asthma and the asthma‐related intestinal metabolites [221]. The South African Food Allergy Cohort was studied to examine dietary factors associated with allergy in rural and urban children (1‐3 years old) and found that consumption of fast food at least once per week was associated with higher rates of AD in urban children. In contrast, data from the same cohort showed that consumption of fermented milk (amasi) was protective and associated with lower rates of AD, asthma, and allergy in the urban cohort but had no effect in the rural cohort. No data were available on microbiome composition [222]. As proposed in several theories linking allergies and exposure to microbes, fermented food might provide an alternative source of environmental microbes and microbial metabolites for children living in an urban area, mitigating allergic disease risk. In adults, increased consumption of fermented foods has been shown to reduce inflammation [223]. In the same African cohort, increased daily intake of sugar and saturated fats was associated with lower diversity of the gut microbiome and decreased relative abundance of *P. copri*. Infants with AD also had lower *P. copri* [224] levels. *P. copri* is believed to confer protective effects against food allergies. Interestingly, maternal carriage of *P. copri* during pregnancy strongly predicted the absence of food allergy in offspring [70, 225]. These data suggest that the microbiome could mediate some of the effect of ‘unhealthy’ foods (processed meat and high daily intake of simple sugars and saturated fat) on infant health, in particular for immune disorders.

## 5 Limitations

While animal models have been instrumental in highlighting the causative role of the microbiome in host physiology, identifying microbiome‐mediated mechanisms in healthy development, and informing the development of microbiota‐based or ‐focused therapeutics, translational progress is still slow. Mice and humans differ significantly in gastrointestinal anatomy, immune system maturation, diet, housing environments, and gut transit time, all of which influence microbial composition and function [226]. For example, coprophagy, on the one hand, and controlled housing and diet, on the other, contribute to a murine gut ecosystem that is markedly different from that of humans. Consequently, these differences affect vertical microbial transmission, immune priming, and early‐life colonization dynamics, complicating the extrapolation of findings to clinical settings.

Similarly, while observational studies in humans provide valuable insights, causal inference from cohort studies is limited due to confounding factors such as diet, medication use, and unmeasured lifestyle variables. In addition, taxonomic profiles are not always shared across populations, limiting generalizability. Shifting to focus on microbial functional potential or metabolic activity can improve research interpretability. Moreover, variations in fecal sampling or processing methods can significantly affect microbiome profiles, and use of this sample substrate may not fully capture mucosal microbiota, which are more directly involved in and associated with host‐microbe interactions than fecal communities, thus potentially masking relevant host‐microbe interactions. Progress in this field depends on integrating multi‐omic datasets with a variety of mechanistic experimental models and clinically and geographically diverse cohorts. Iterative, hypothesis‐driven studies designed with multiple checks and balances in place [226, 227] remain critical for furthering our understanding of how maternal and neonatal nutrition influence early‐life microbiota programming.

Lastly, as this is a narrative review, it does not follow a systematic review methodology and may be subject to selection bias. We conducted a targeted review of the literature, with preference given to peer‐reviewed English‐language publications from the past 10 years, alongside the inclusion of seminal earlier studies (see Table 1 for search terms). While this approach allows for broad thematic synthesis, conclusions should be interpreted within the context of these methodological constraints.

## 6 Conclusion and Perspectives

The studies described here highlight how nutritional state, in both the mother and neonate, can shape the microbiome of infants and children. The microbiome is primarily shaped in the first 2‐3 years of life, as newborns are exposed to increasing diversity of environments, foods, and people. Diet has significant effects on the initiation and stabilization of the microbial community. In addition, some of these studies suggest that the gut microbiome, when it is disturbed, could be one of the elements feeding the vicious intergenerational cycle of malnutrition [25, 186].

The first years of life are a unique window in which to build and preserve a healthy, supportive, symbiotic relationship between gut microbes and the host. Because the infant microbiome is also highly malleable during this period, there are opportunities for microbiome‐based or ‐targeted approaches in addition to prevention strategies to improve later health outcomes.

Diet is one of the primary factors influencing the gut microbiome in prenatal and postnatal periods. Long‐term adherence to a microbiome‐friendly, healthy diet might be the most accessible approach to prevent chronic NCDs and promote health. A healthy diet starts with breastfeeding. WHO and UNICEF recommend exclusive breastfeeding for the first 6 months of life and continued breastfeeding up to 2 years of age and beyond. Data based on microbiome research support this recommendation, showing that breastfeeding might be the best preventive health practice for infants and might be one of the best restorative measures naturally available after C‐section and for preterm babies. But despite the many benefits of EBF, it is not the norm in many countries. The promotion, support, and protection of optimum breastfeeding requires a different type of engagement from the health system. Major challenges limit optimal breastfeeding practices, among them the belief that infants need water in addition to HM or that cow's milk and HM are comparable and a lack of support for breastfeeding at home, in the community, in health care facilities, and in workplaces. A systematic review of infant formula trials recently showed that formula trials lack independence or transparency and that published outcomes are biased by selective reporting [228], which could promote formula milk to the detriment of breastfeeding. On the whole, adding the dimension of microbiota health could deepen the conversation around breastfeeding, providing more support for its benefits across cultures.

The microbiota should be considered a key component of nutrition education and public health messages, and future dietary recommendations should take into account not only human nutritional requirements but also those of the gut microbiota [229]. Special attention to the nutrition of pregnant women appears to be important to best prime the infant microbiome and improve maternal and child health outcomes. Studies support the notion that teaching women to ‘eat for their gut microbiota’ may be effective in motivating and empowering them to increase their diet quality and variety, for example, by focusing on dietary fiber, fermented foods, and plant‐based foods [229, 230, 231], as well as prebiotic and probiotic foods, at least in the short term [232].

Developing access to healthy and sustainable diets that take the microbiome into consideration requires holistic and coordinated actions, with policy implications on health and agrifood systems [233]. Furthermore, effects of food additives and exogenous compounds found in food (i.e., pesticide residues, antimicrobials, or microplastics) on the maternal and neonatal microbiome and metabolome should be evaluated as part of more general risk assessments of food safety, as the limited available preliminary data suggest that they can disturb the microbiome [234]. The consequences of exogenous compounds on the microbiome and health, if validated, might have an even stronger effect during pregnancy and in childhood than during other times of life.

In addition, prophylactics to modulate or enhance microbial colonization could be relevant, for example, in infants who were born via C‐section or not exclusively breastfed, but stronger evidence is needed before interventions can be recommended. In the future, personalized dietary recommendations for mother and child could be made based on birth mode, feeding choices, antibiotic administration, and other early life parameters. In adults, data suggest that a personalized diet integrating individual microbiome composition and function could promote health [235, 236, 237]; to our knowledge, no such studies have been performed in children.

In conclusion, the microbiome integrates the effect of multiple environmental factors and therefore serves as a readout of the health and nutritional state of the individual. Overall data on microbiome development support existing public health recommendations regarding maternal and child health and nutrition, and further study of microbiota‐derived health consequences could help to improve them.

## Disclosure

The views expressed in this publication are those of the authors and do not necessarily reflect the views or policies of the Food and Agriculture Organization of the United Nations.
